# IncL plasmid-mediated dissemination of OXA-48 β-lactamase and *bla*_CTX-M-15_ gene amplification identified *via* long-read sequencing in carbapenem-resistant Enterobacterales

**DOI:** 10.1093/jacamr/dlaf254

**Published:** 2026-01-07

**Authors:** Suhanya Prasad, Barbora Dratvova, Anezka Gryndlerova, Marie Brajerova, Petra Kabelikova, Jan Tkadlec, Pavel Drevinek, Marcela Krutova

**Affiliations:** Department of Medical Microbiology, Second Faculty of Medicine, Charles University, and Motol University Hospital, V Uvalu 84, Praha 5, Prague 150 06, Czech Republic; Department of Medical Microbiology, Second Faculty of Medicine, Charles University, and Motol University Hospital, V Uvalu 84, Praha 5, Prague 150 06, Czech Republic; Department of Medical Microbiology, Second Faculty of Medicine, Charles University, and Motol University Hospital, V Uvalu 84, Praha 5, Prague 150 06, Czech Republic; Department of Medical Microbiology, Second Faculty of Medicine, Charles University, and Motol University Hospital, V Uvalu 84, Praha 5, Prague 150 06, Czech Republic; Department of Medical Microbiology, Second Faculty of Medicine, Charles University, and Motol University Hospital, V Uvalu 84, Praha 5, Prague 150 06, Czech Republic; Department of Medical Microbiology, Second Faculty of Medicine, Charles University, and Motol University Hospital, V Uvalu 84, Praha 5, Prague 150 06, Czech Republic; Department of Medical Microbiology, Second Faculty of Medicine, Charles University, and Motol University Hospital, V Uvalu 84, Praha 5, Prague 150 06, Czech Republic; Department of Medical Microbiology, Second Faculty of Medicine, Charles University, and Motol University Hospital, V Uvalu 84, Praha 5, Prague 150 06, Czech Republic

## Abstract

**Background:**

Increasing resistance to broad-spectrum beta-lactams and carbapenems is a significant concern in healthcare settings. This study aimed to determine the prevalence of intestinal carriage of extended-spectrum β-lactamase (ESBL)-producing and carbapenem-resistant Enterobacterales (CRE) in a tertiary care hospital and to evaluate the utility of long-read sequencing for carbapenem resistance surveillance.

**Methods:**

In 2021, stool samples (*n* = 538) and rectal swabs (*n* = 256) from hospitalized patients were cultured after enrichment on selective chromogenic medium to detect ESBL and CRE carriage. CRE isolates were characterized by antimicrobial susceptibility testing and whole-genome sequencing.

**Results:**

Among 794 patient samples, 239 (30%) Enterobacterales isolates grew on ESBL media. On CRE agar, 28 Enterobacterales were cultured, 27 confirmed carbapenem-resistant and identified as *Klebsiella pneumoniae* (*n* = 25), *Escherichia coli* (*n* = 1), and *Enterobacter cloacae* (*n* = 1). In CRE, 29.6% (8/27) were carbapenemase-producing Enterobacterales (CPE), carrying the *bla*_OXA-48_ (*n* = 7) or *bla*_NDM-1_ (*n* = 1) genes. The remaining 70.4% (19/27) were non-carbapenemase-producing CRE isolates (non-CP-CRE). The *bla*_OXA-48_ gene was localized on identical IncL plasmids with an inverted Tn*1999.2* transposon in non-clonally related isolates. CPE isolates exhibited distinct resistance patterns to carbapenems, β-lactam/β-lactamase inhibitor combinations, with 87.5% resistant to cefiderocol. All non-CP-CRE isolates remained susceptible to imipenem; two were resistant to meropenem and carried either five or six copies of the *bla*_CTX-M-15_ gene along with mutations in porin genes.

**Conclusions:**

A 30% prevalence of intestinal carriage of ESBL-producing Enterobacterales and a 3.4% carriage prevalence of CRE were found. Long-read sequencing revealed IncL plasmid-mediated dissemination of OXA-48 β-lactamase and *bla*_CTX-M-15_ gene amplification, demonstrating its added value for antimicrobial resistance monitoring.

## Introduction

Increasing resistance to broad-spectrum beta-lactams and carbapenems is a significant concern in healthcare settings. In addition, carbapenem-resistant Enterobacterales (CRE) infections are associated with high morbidity and mortality, particularly in patients with comorbidities, and treatment options remain severely limited.^1,2^

Carbapenem resistance in Enterobacterales is primarily mediated by plasmid-encoded carbapenemase enzymes, which enable their rapid spread among bacterial species. These plasmids often co-carry additional antimicrobial resistance genes, contributing to multidrug resistance against cephalosporins, aminoglycosides, sulphonamides, and quinolones.^3–5^

Non-carbapenemase-producing CRE (non-CP-CRE) typically develop resistance through alternative mechanisms, such as porin inactivation and efflux pump overexpression, which reduce antibiotic concentration in the periplasmic space.^6^ Although extended-spectrum β-lactamases (ESBLs) do not confer carbapenem resistance directly, growing evidence indicates that increased gene copy number, particularly in combination with porin loss, can contribute to reduced susceptibility to carbapenems.^2,7,8^

This study aimed to determine the prevalence of intestinal carriage of ESBL-producing and or CRE in a tertiary care hospital and to investigate the genetic mechanisms underlying carbapenem resistance.

## Materials and methods

### Samples, culture, species identification and antimicrobial susceptibility testing

A total of 794 samples were collected from hospitalized patients at Motol University Hospital, Prague, Czech Republic, between January and October 2021. These included stool samples (*n* = 538) from patients with diarrhoea and rectal swabs from patients screened for intestinal carriage of multidrug-resistant bacteria (*n* = 256). To reflect the overall patient population in the hospital, samples were randomly selected across different hospital departments, excluding duplicate specimens from the same patient.

Samples were first enriched in Enterobacteriaceae Enrichment Broth Mossel (Oxoid, UK) overnight and then cultured on a selective chromogenic media for the detection of ESBL-producing (CHROMagar^™^ Orientation with 0.57 g/L ESBL supplement ES372, France) and carbapenem-resistant [CHROMagar^™^ Orientation with 0.4 g/L *Klebsiella pneumoniae* carbapenemase (KPC) supplement, France] Enterobacterales. Bacterial species were identified using Matrix Assisted Laser Desorption/Ionization-Time of Flight mass spectrometry on Biotyper v 3.1 (Bruker Daltonics, USA).

In CRE isolates, antimicrobial susceptibility testing (AST) to 24 antibiotics was performed using the broth microdilution method (Erba Lachema, Germany), with imipenem Minimum Inhibitory Concentration (MICs) determined by MIC test strip (Liofilchem, Italy). AST results were interpreted according to the European Committee on Antimicrobial Susceptibility Testing (EUCAST) breakpoints (v13.0). Clinical and Laboratory Standards Institute (CLSI) breakpoints were applied for cefoperazone, netilmicin and tetracycline.^9,10^

Additionally, in carbapenemase-producing Enterobacterales (CPE) isolates, susceptibility to novel β-lactam-β-lactamase inhibitor combinations (βL-βLIs), including meropenem–vaborbactam, imipenem–relebactam, ceftazidime–avibactam and ceftolozane–tazobactam, was determined using the MIC test strip (bioMérieux, France). Susceptibility to cefiderocol was tested by the disc diffusion (Oxoid, UK) and to fosfomycin using the MIC test strip (Liofilchem, Italy). Results were interpreted according to the EUCAST breakpoints (v15.0) and/or epidemiological cut-offs (ECOFFs).^11^

### Whole-genome sequencing and bioinformatic analysis

Short-read sequencing was performed for all the CRE isolates. Genomic DNA was extracted using the MasterPure Complete DNA and RNA Purification Kit (BioSearch Technologies, UK). Library preparation was performed using the Nextera XT DNA Library Preparation Kit and sequenced on the NovaSeq6000 platform (Illumina, USA). Raw reads were assembled using SPAdes v3.15.5,^12^ and genome annotation was performed with RASTtk.^13^

All CPE isolates underwent long-read sequencing. Among the non-CP-CRE isolates, long-read sequencing was performed on six isolates selected based on specific phenotypic or genomic characteristics. This included two isolates resistant to meropenem, one isolate with a lower meropenem MIC used as a comparator and three isolates in which short-read assemblies failed to recover the *ompK36* or *ompC* gene on one contig. DNA libraries were prepared using either the Ligation Sequencing Kit (SQK-LSK109), the Native Barcoding Kit (SQK-NBD114.24) or the Rapid Barcoding Kit (SQK-RBK114.24) and sequenced on the MinION device using R9.4.1 or R10.4.1 flow cell (both from Oxford Nanopore Technologies, UK). Hybrid assembly was performed using Flye assembler v2.9.4,^14^ Medaka v2.0.1 (polishing by long reads) (Oxford Nanopore Technologies, UK) and Polypolish v0.6.0 (polishing by short reads).^15^

Antimicrobial resistance determinants (genes and/or chromosomal mutations) were searched using ResFinder v4.5,^16^ available at: https://www.genomicepidemiology.org. Mutations in genes encoding porins (*ompK*35/*ompF*, *ompK*36/*ompC*, and *ompK*37/*ompN*) and the penicillin-binding proteins (PBPs), such as *mrdA* (PBP2) and *ftsI* (PBP3), associated with carbapenem resistance were investigated by aligning raw reads to the reference genomes of *Klebsiella pneumoniae* ATCC 13883 (NZ_KN046818), *Escherichia coli* K-12 substr. MG1655 (NC_000913) and *Enterobacter cloacae* ATCC 13047 (NC_014121) using Snippy v4.6.0.^17^ The *ompK*37 gene in *E. cloacae* complex was excluded from comparative analysis due to the limited data on its relevance.

Suspected cefiderocol resistance mechanisms were assessed by analysing siderophore receptor genes (*cirA*, *fiu*, *fhuA*, *efeO*, *exbD*) using *K. pneumoniae* ATCC 13883 (NZ_KN046818) as the reference genome. Additionally, mutations in genes associated with resistance to colistin (*mgrB* and *phoPQ*) and tigecycline (*ramR*, *oqxR* and *acrA*) were searched through sequence alignment with the wild-type reference *K. pneumonia*e ATCC 13883 (NZ_KN046818) and MGH78578 (CP000647) using MUltiple Sequence Comparison by Log-Expectation (MUSCLE) aligner in Geneious Prime 2024.0.5 (Dotmatics, USA) and confirmed using Snippy v4.6.0.^17^

*In silico* Multilocus sequence typing (MLST) was determined using MLST 2.0.^18^ The wgMLST analysis of carbapenem-resistant *K. pneumoniae* (*n* = 25) was performed using Bionumerics v8.1, analysing 7764 loci (bioMérieux, France). Clonal relatedness was defined as ≤10 allele differences.^19^ Plasmid replicons were identified using the PlasmidFinder v2.1.^20^

### Plasmid comparative analysis

To analyse the genetic background and relatedness of plasmids carrying carbapenemase genes, the IncL plasmids harbouring the *bla*_OXA-48_ gene, encoding oxacillinase, (*n* = 7) were compared with the prototype plasmid pOXA-48a (JN626286.1) from *K. pneumoniae*,^21^ the pRA35 plasmid (LN864821.1) from *Raoutella planticola*, which initially described the inverted Tn*1999.2* transposon variant,^22^ and representative plasmids from a previous OXA-48-like associated hospital outbreak in the Czech Republic.^23^ For standardization, all plasmids were reannotated in Geneious Prime v2024.0.5, and their sequences were reordered to start at the *rep*A gene using Circlator v1.5.5.^24^

The sequence of the New Delhi metallo-β-lactamase-1 (NDM-1) carrying IncFIB(pQil)/IncFII(K) plasmid was searched in National Center for Biotechnology Information (NCBI) *via* Basic Local Alignment Search Tool for Nucleotides (BLASTn), and the top three matches were selected for comparative genomic analysis. Visualization was performed using Easyfig v2.2.5 and BRIG v0.95,^25,26^ with final figure refinement performed in Inkscape v1.3.2.

## Results

From 794 non-duplicated samples, 239 of them (30%) were culture positive on ESBL-selective culture media, yielding *K. pneumoniae* (*n* = 107), *E. coli* (*n* = 56), *E. cloacae* complex (*n* = 48), and other bacterial species (*n* = 28). Further analysis was not performed on these isolates.

On CRE selective culture media, 28 Enterobacterales were cultured and 27 confirmed resistant to carbapenems by AST, and identified as *K. pneumoniae* (*n* = 25), *E. coli* (*n* = 1), and *E. cloacae* (*n* = 1). The overall CRE prevalence was 3.4% [95% Wilson score confidence interval (CI) 2.35%–4.90%]. Of the 27 isolates, 9 originated from patients in standard wards and 18 from patients in intensive care units. All isolates were classified as colonization. Antibiotic therapy had been administered to 26 patients, and 55.6% (*n* = 15/27) had received carbapenem treatment within 1 month prior to sampling.

**Figure 1.**
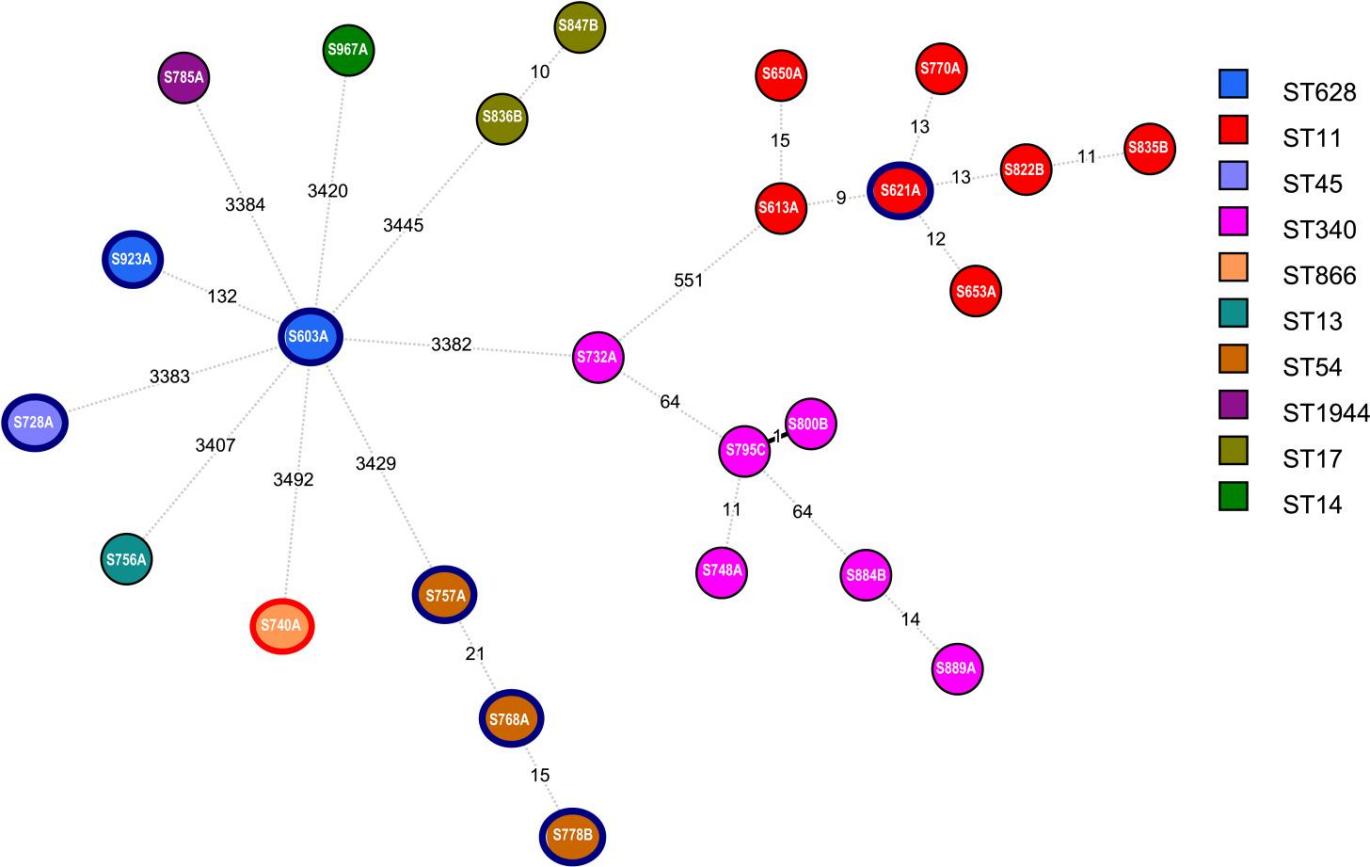
Minimum spanning tree based on the wgMLST analysis of carbapenem-resistant *K. pneumoniae* isolates (*n* = 25, Bionumerics v8.1, 7764 loci). The numbers on the branches between isolates indicate the number of allele differences. Blue and red outer rings highlight OXA-48- and NDM-1-carrying isolates, respectively.

The *K. pneumoniae* isolates belonged to 10 different sequence types (STs), with the high-risk clone ST11 being the most prevalent (28%, *n* = 7/25), and ST340 was the second most common (24%, *n* = 6/25). The *E. coli* and *E. cloacae* isolates belonged to ST131 and ST764, respectively. The wgMLST analysis showed allelic differences ranging from 1 to 3492. Clonal relatedness with ≤10 allele differences was only identified between two isolates in the following STs: ST17, ST11 and ST340 (Figure 1).

Among the 27 ertapenem-resistant isolates, eight (29.6%) were carbapenemase producers harboured either the *bla*_OXA-48_ (*n* = 7) or *bla*_NDM-1_ (*n* = 1) genes; CPE prevalence of 1% (95% CI 0.51%–1.98%). The *bla*_NDM-1_ gene-carrying isolate exhibited a resistant phenotype, with MICs above the clinical breakpoints for all carbapenems, imipenem/relebactam, ceftazidime/avibactam and ceftolozane/tazobactam (Table 1), and carried mutations in the porin *ompK37* and the *mrdA* (PBP2) gene. Two OXA-48 producers (S603A, S923A) were resistant to all three tested carbapenems and to imipenem/relebactam, and carried multiple missense mutations in *ompK36*, including A217S and N218H substitutions (identified by ResFinder), and a frameshift mutation (N335fs). They also carried a missense mutation in *ompK35* (E132K), which had been previously reported in meropenem- and meropenem–vaborbactam-resistant strains;^27^ however, both strains in our study were borderline susceptible to meropenem/vaborbactam (8 mg/L). Four OXA-48-producing isolates (S728A, S757A, S768A and S778B) carried a missense substitution (V178P) in *ompK36*, previously reported in a multidrug-resistant *K. pneumoniae*;^28^ two of these (S757A and S768A) were resistant to all carbapenems. Additional missense substitutions were found, including G191T, F200Y, I312L and L320I (*n* = 5) and Y209W, R355N and T256S (*n* = 2), although these variants were previously reported in ertapenem- and meropenem-susceptible isolates (Table 1).^29^ Missense and/or frameshift mutations in *ompK37* were predicted by ResFinder in all isolates.

**Table 1.** Susceptibility testing of carbapenems and βL/βLI combinations, and characterization of resistance mechanisms in carbapenemase-producing isolates (*n* = 8)

Isolate ID	OXA-48/NDM-1	ERT (>0.5 mg/L)	IMI (>4 mg/L)	MER (>8 mg/L)	I/R (>2 mg/L)	M/V (>8 mg/L)	ATM–AVI (4 mg/L)	CZA- AVI (>8 mg/L)	C/T (>2 mg/L)	Potential acquired resistance genes and mutations						
										*ompK*35	*ompK*36^a^	*ompK*37	*mrdA* (PBP2)	*ftsI* (PBP3)	ESBLs (n)	Other β-lactamases
S603A	OXA-48	**4**	**8**	**32**	**32**	8	0.047	0.25	**16**	E132K	A217S, N218H, N335fs, (Y209W, R355N, T256S)	I128M, I70M, N230G, M233fs	Q509P, D461A, A274V, S223T	WT	*bla*_CTX-M-15_ (1)	*bla*_SHV-1_, *bla*_OXA-1_, *bla*_TEM-1B_
S621A	OXA-48	**4**	1	4	0.38	0.38	0.094	0.75	**>256**	WT	(G191T, F200Y, I312L, L320I)	I128M, I70M, N230G, M233fs	P97H, Q509P, D461A, A274V, S223T	WT	*bla*_CTX-M-15_ (4)	*bla*_SHV-11_, *bla*_OXA-1_, *bla*_TEM-1B_
S728A	OXA-48	**1.5**	4	4	1	0.75	0.047	0.38	**16**	WT	V178P, (G191T, F200Y, I312L, L320I)	I128M, I70M, N230G, M233fs	Q509P, D461A, A274V, S223T	WT	*bla*_CTX-M-15_ (1)	*bla*_SHV-1_, *bla*_OXA-1_, *bla*_TEM-1B_
S757A	OXA-48	**>32**	**12**	**32**	**32**	**>64**	0.25	1	**48**	WT	V178P, (G191T, F200Y, I312L, L320I)	I128M, I70M, N230G, M233fs	V276L, A633V, Q509P, D461A, A274V, S223T	WT	*bla*_CTX-M-15_ (2)	*bla*_SHV-11_, *bla*_OXA-1_, *bla*_TEM-1B_
S768A	OXA-48	**>32**	**16**	**32**	**32**	**32**	0.19	1	**64**	WT	V178P, (G191T, F200Y, I312L, L320I)	I128M, I70M, N230G, M233fs	V276L, A633V, Q509P, D461A, A274V, S223T	WT	*bla*_CTX-M-15_ (2)	*bla*_SHV-11_, *bla*_OXA-1_, *bla*_TEM-1B_
S778B	OXA-48	**8**	1.5	**32**	1.5	1.5	0.19	0.5	**24**	WT	V178P, (G191T, F200Y, I312L, L320I)	I128M, I70M, N230G, M233fs	V276L, Q509P, D461A, A274V, S223T	WT	*bla*_CTX-M-15_ (2)	*bla*_SHV-11_, *bla*_OXA-1_, *bla*_TEM-1B_
S923A	OXA-48	**3**	**24**	**32**	**>32**	8	0.094	0.25	**16**	E132K	A217S, N218H, N335fs (Y209W, T256S, R355N)	I128M, I70M, N230G, M233fs	Q509P, D461A, A274V, S223T	WT	*bla*_CTX-M-15_ (1)	*bla*_SHV-1_, *bla*_OXA-1_, *bla*_TEM-1B_
S740A	NDM-1	**8**	**6**	**32**	**6**	3	0.125	**>256**	**>256**	WT	WT	I128M, I70M	Q509P, D461A, A274V, A22S, S223T	WT	*bla*_CTX-M-15_ (2)	*bla*_SHV-11_, *bla*_OXA-1_, *bla*_OXA-9_, *bla*_TEM-1B_, *bla*_TEM-1A_

MIC values in bold indicate ‘resistant’ categorization. Underlined indicates that substitution at this position has been reported previously in resistant isolates or predicted using ResFinder v4.5. The number within brackets denotes the copies of the *bla*_CTX-M-15_ gene. Mutations within the brackets were reported previously.

ERT, ertapenem; IMI, imipenem; MER, meropenem; ATM/AVI, aztreonam/avibactam; CZA/AVI, ceftazidime/avibactam; C/T, ceftolozane/tazobactam; I/R, imipenem/relebactam; M/V, meropenem/vaborbactam; WT, wild-type; fs, frameshift.

^a^All Missense and frameshift mutations are presented.

Two OXA-48 producers (S757A and S768A) were resistant to meropenem/vaborbactam and carried missense mutations in *mrdA* gene (V276L and A633V). These mutations were not detected in any of the other isolates, except for one OXA-48 producer (S778B), which was resistant to ertapenem and meropenem and carried the substitution (V276L) in *mrdA*. The remaining two OXA-48 producers were resistant only to ertapenem; of these, isolate S621A carried the P97H mutation in *mrdA* and exhibited a higher MIC to ceftolozane/tazobactam than the other OXA-48 producers. However, the P97H substitution was also detected in non-CP-CRE isolates; their susceptibility to ceftolozane/tazobactam was not tested.

Cefiderocol resistance was observed in 7/8 CPE isolates, with three isolates falling in the area of technical uncertainty (ATU, 21–23 mm). All eight isolates carried one or more missense mutations in the siderophore receptor genes (*cirA*, *fiu*, *fhuA*, *efeO* and *exbD*), including mutations in *cirA* (A134V and N558D) and *fiu* (A32T, A287T, D387N and S718R) genes that had been previously associated with cefiderocol resistance.^30^ Although all CPE isolates were phenotypically wild-type for fosfomycin (MIC ≤ 128 mg/L), they carried the *fosA5* or *fosA6* genes on the chromosome (Table 2).

**Table 2.** Susceptibility testing of cefiderocol and fosfomycin, and characterization of resistance mechanisms in carbapenemase-producing isolates (*n* = 8)

Isolate ID	CFC^a^ (<23 mm)	FSF (^b^128 mg/L)	Potential acquired resistance genes and mutations					
			*cirA*	*fiu*	*fhuA*	*efeO*	*exbD*	FosA variants
S603A	23	16	A134V, N558D, Y183W, D537Y	A287T	N421S	Q45R	WT	FosA6 (99% identity and 100% coverage; AMQ12811.1), FosA7-like (88% identity and 59% coverage; QJH46864.1)
S621A	**22**	3	A134V, N558D	A32T, A287T, S718R	F735Y	WT	WT	FosA6 (98% identity and 100% coverage; AMQ12811.1)
S728A	**16**	8	A134V, N558D, V237I	S718R	WT	WT	WT	FosA5 (100% identity and coverage; XVW23973.1)
S757A	**15**	32	A134V, N558D	A287T, A663G, E231K, D387N	WT	A171T	T94A	FosA5 (100% identity and coverage; XJT18122.1)
S768A	**14**	48	A134V, N558D	A287T, A663G, E231K, D387N	WT	A171T	T94A	FosA5 (100% identity and coverage; XJT18122.1)
S778B	**16**	12	A134V, N558D	A287 T, A663G, E231K, D387N	WT	A171T	T94A	FosA5 (100% identity and coverage; XJT18122.1)
S923A	**22**	24	A134V, N558D, Y183W, D537Y	A287T	N421S	Q45R	WT	FosA6 (99% identity, 100% coverage; AMQ12811.1), FosA7-like (87% identity and 60% coverage; QJH46864.1)
S740A	**11**	4	A134V	S718R, D387N	A48T, A50V	WT	WT	FosA5 (100% identity and coverage; XAY83656.1)

Underlined mutations have been published previously as being associated with cefiderocol resistance.

CFC, cefiderocol; FSF, fosfomycin.

^a^Disk diffusion (30 µg).

^b^ECOFF. MIC values in bold indicate ‘resistant’ categorization.

The remaining 19 (70.4%) ertapenem-resistant isolates were non-CP-CRE, with a prevalence of 2.4% (95% CI 2.35%–4.90%). Among them, two isolates were also resistant to meropenem, with MICs of 12 mg/L (Table 3). Non-CP-CRE isolates showed either loss-of-function (premature stop codon/deletion) or frameshift and missense mutations in the *ompK36* gene. In one isolate (S770A), the *ompK36* gene was absent. An IS5-like insertion element from the IS903B transposase family disrupted *ompK36* in two *K. pneumoniae* isolates (S795C, S800B). In the *E. coli* isolate S931B, an IS26-linked composite transposon (cn_3654_IS26) carrying IS26-IS5 (IS903B family)-*bla*_CTX-M-27_-IS26 was inserted in the *ompC* gene, disrupting its coding sequence and likely impairing porin function (seen in hybrid assemblies). The summary of MICs and resistance mechanisms is provided in Table 3. Porin modifications, comprising insertions and deletions (indels), and complex substitutions are listed in Table S3 (available as Supplementary data at *JAC-AMR* Online).

**Table 3.** Carbapenem susceptibility and resistance mechanisms in non-carbapenemase-producing isolates (*n* = 19)

Isolate ID	Species	ERT (>0.5 mg/L)	IMI (>4 mg/L)	MER (>8 mg/L)	Potential acquired resistance genes and mutations						
					*ompK*35	*ompK*36^a^	*ompK*37	ESBLs (n)	other β-lactamases	*mrdA* (PBP2)	*ftsI* (PBP3)
S613A	*K. pneumoniae*	**>32**	4	**12**	WT	A217S, N218H, (G191T, F200Y, I312L, L320I)	I128M, I70M, N230G, M233fs	*bla*_CTX-M-15_ (5)	*bla*_SHV-11_, *bla*_OXA-1_, *bla*_TEM-1B_	P97H, Q509P, D461A, A274V, S223T	WT
S650A	*K. pneumoniae*	**>32**	1.5	8	WT	G73fs, (G191T, F200Y, I312L, L320I)	I128M, I70M, N230G, M233fs	*bla*_CTX-M-15_	*bla*_SHV-11_, *bla*_OXA-1_, *bla*_TEM-1B_	P97H, Q509P, D461A, A274V, S223T	P305L
S653A	*K. pneumoniae*	**>32**	2	**12**	WT	Q170^b^	I128M, I70M, N230G, M233fs	*bla*_CTX-M-15_ (6)	*bla*_SHV-11_, *bla*_OXA-1_, *bla*_TEM-1B_	P97H, Q509P, D461A, A274V, S223T	WT
S732A	*K. pneumoniae*	**3**	0.5	4	WT	(G191T, F200Y, I312L, L320I)	I128M, I70M, N230G, M233fs	*bla*_CTX-M-15_	*bla*_SHV-11_, *bla*_OXA-1_, *bla*_TEM-1B_	P97H, Q509P, D461A, A274V, S223T	WT
S748A	*K. pneumoniae*	**>32**	0.75	2	WT	(G191T, F200Y, I312L, L320I)	I128M, I70M, N230G, M233fs	*bla*_CTX-M-15_, *bla*_SHV-12_	*bla*_SHV-11_, *bla*_OXA-1_, *bla*_TEM-1B_	P97H, Q509P, D461A, A274V, S223T	WT
S756A	*K. pneumoniae*	**>32**	0.5	4	WT	E272^b^	I128M, I70M	*bla*_CTX-M-15_	*bla*_SHV-1_, *bla*_OXA-1_, *bla*_TEM-1B_	E407A, L251I, Q509P, D461A, A274V, S223T	WT
S770A	*K. pneumoniae*	**>32**	1	2	WT	Gene deletion	I128M, I70M, N230G, M233fs	*bla*_CTX-M-15_	*bla*_SHV-11_, *bla*_OXA-1_, *bla*_TEM-1B_	P97H, Q509P, D461A, A274V, S223T	WT
S785A	*K. pneumoniae*	**6**	1.5	2	WT	WT	I128M, I70M	—	*bla*_SHV-11_, *bla*_LAP-2_	Q509P, D461A, A274V, S223T	WT
S795C	*K. pneumoniae*	**>32**	1.5	2	WT	IS903B insertion	I128M, I70M, N230G, M233fs	*bla*_CTX-M-15_, *bla*_SHV-12_	*bla*_SHV-11_, *bla*_OXA-1_, *bla*_TEM-1B_	P97H, Q509P, D461A, A274V, S223T	WT
S800B	*K. pneumoniae*	**1**	0.25	2	WT	IS903B insertion	I128M, I70M, N230G, M233fs	*bla*_CTX-M-15_, *bla*_SHV-12_	*bla*_SHV-11_, *bla*_OXA-1_, *bla*_TEM-1B_	P97H, Q509P, D461A, A274V, S223T	WT
S822B	*K. pneumoniae*	**>32**	1	2	WT	Q76^b^	I128M, I70M, N230G, M233fs	*bla*_CTX-M-15_ (4)	*bla*_SHV-11_, *bla*_OXA-1_, *bla*_TEM-1B_	P97H, Q509P, D461A, A274V, S223T	WT
S835B	*K. pneumoniae*	**>32**	1	4	WT	Q76^b^	I128M, I70M, N230G	*bla*_CTX-M-15_	*bla*_SHV-11_, *bla*_OXA-1_, *bla*_TEM-1B_	P97H, Q509P, D461A, A274V, S223T	WT
S836B	*K. pneumoniae*	**>32**	1.5	4	L14V	L8fs, (L193Q, L320I, R355N)	I128M, I70M	*bla*_CTX-M-15_	*bla*_SHV-11_, *bla*_OXA-1_, *bla*_TEM-1B_	Q509P, D461A, A274V, S223T	WT
S847B	*K. pneumoniae*	**2**	0.75	0.5	L14V	L8fs, (L193Q, L320I, R355N)	I128M, I70M	*bla*_CTX-M-15_	*bla*_SHV-11_, *bla*_OXA-1_, *bla*_TEM-1B_	Q509P, D461A, A274V, S223T	WT
S884B	*K. pneumoniae*	**>32**	1	8	WT	Partial gene deletion (459 bp)	I128M, I70M, N230G, M233fs	*bla*_CTX-M-15_	*bla*_SHV-11_, *bla*_OXA-1_, *bla*_TEM-1B_	P97H, Q509P, D461A, A274V, S223T	WT
S889A	*K. pneumoniae*	**>32**	1	4	WT	Partial gene deletion (459 bp)	I128M, I70M, N230G, M233fs	*bla*_CTX-M-15_	*bla*_SHV-11_, *bla*_OXA-1_, *bla*_TEM-1B_	P97H, Q509P, D461A, A274V, S223T	WT
S967A	*K. pneumoniae*	**6**	1.5	2	WT	A217S, N218H, (Y209W, L320I, R355N)	I128M, I70M	—	*bla*_SHV-1_	P512A, Q509P, D461A, A274V, S223T	WT
S931B	*E. coli*	**>32**	1.5	4	S226P	IS26 insertion	K90T	*bla*_CTX-M-27_	*bla*_TEM-1B_	WT	A233T, I332V
P2697A	*E. cloacae*	**6**	1	2	V141A	S155Q, A177P, D181G, T188D,E189A, S223D, N232E	n/a	—	*bla*_ACT-14_	E103N, N112T, N347T, V274I, V419I, E490D, F511Y, R563K	R2K, R152K, H162R, E378D, V440I, I458 V,V563I

MIC values in bold indicate ‘resistant’ categorization. Underlined indicates that substitution at this position has been predicted using ResFinder v4.5. The substitutions within brackets indicate those reported previously in susceptible isolates. The number within brackets denotes the copies of the bla_CTX-M-15_ gene.

fs, frameshift; WT, wild-type; —, not found; n/a, not applicable.

^a^All missense, frameshift, and nonsense mutations are presented.

^b^Stop codon.

Among the non-CP-CRE isolates, we compared the genomes of two meropenem-resistant ST11 isolates (S613A and S653A) with a meropenem-susceptible isolate (S822B) using whole-genome alignment to identify resistance-related differences. S613A carried five copies of *bla*_CTX-M-15_ (four chromosomal and one plasmid). S653A carried six copies of *bla*_CTX-M-15_ (five chromosomal and one plasmid). The meropenem-susceptible S822B carried four copies of *bla*_CTX-M-15_ (three chromosomal and one plasmid). In all three isolates, an IS*1380*-like element (IS*Ecp1*) was located upstream of the *bla*_CTX-M-15_ gene. In the resistant isolates, the extra copies were also contained within the IS*Ecp1*-*bla*_CTX-M-15_ transposition unit.

All isolates were resistant to ampicillin, piperacillin and cephalosporins, including ceftazidime and cefepime, due to the presence of β-lactamase genes. In *K. pneumoniae* isolates, 80% (20/25) carried the *bla*_CTX-M-15_ gene, and in 12% (3/25), a combination of the *bla*_CTX-M-15_ and *bla*_SHV-12_ genes. Co-carriage of the *bla*_OXA-1_ gene was observed in 88% (22/25) of *K. pneumoniae* isolates, with one isolate (S740A) additionally carrying the *bla*_OXA-1_ and *bla*_OXA-9_ genes (Tables 1 and 3). Two *K. pneumoniae* isolates (S785A, S967A) carried narrow β-lactamase genes, *bla*_LAP-2_ or *bla*_SHV-1_, respectively. The *bla*_CTX-M-27_ gene was detected only in the *E. coli* isolate (S931B). *E. cloacae* isolate (P2697A) carried the *bla*_ACT-14_ (*amp*C) gene.

Phenotypic susceptibility data and corresponding molecular resistance mechanisms for non-β-lactam antibiotics are presented in Tables S1 and S2.

### Comparative analysis of the *bla*_OXA-48_ gene-carrying IncL plasmids

IncL plasmids carrying *bla*_OXA-48_ gene were identified in seven *K. pneumoniae* isolates across various STs: ST54 (*n* = 3), ST628 (*n* = 2), ST45 (*n* = 1) and ST11 (*n* = 1), and showed a high sequence similarity of 99.8%, with an approximate length of 63.5 kb and a G + C content of 51.2%. The genetic environment of the *bla*_OXA-48_ gene (798 bp) was identical across all seven plasmids and associated with an inverted variant of Tn*1999.2* transposon. Apart from the *bla*_OXA-48_ gene, no other antimicrobial resistance genes were detected in these plasmids.

Pairwise comparison of plasmids revealed 99.9% sequence identity at 98% coverage with the ancestral plasmid pOXA-48a,^21^ which carries the original variant of Tn*1999* transposon (also referred to as Tn*1999.1*) and 99.9% identity at 100% coverage with pRA35,^22^ which harbours the inverted Tn*1999.2* and Tn*6237* transposons. The 21.9 kb composite transposon Tn*6237* element spans from the first IS*1R* flanking the *bla*_OXA-48_ gene to a second IS*1R* inserted in orf25.

**Figure 2.**
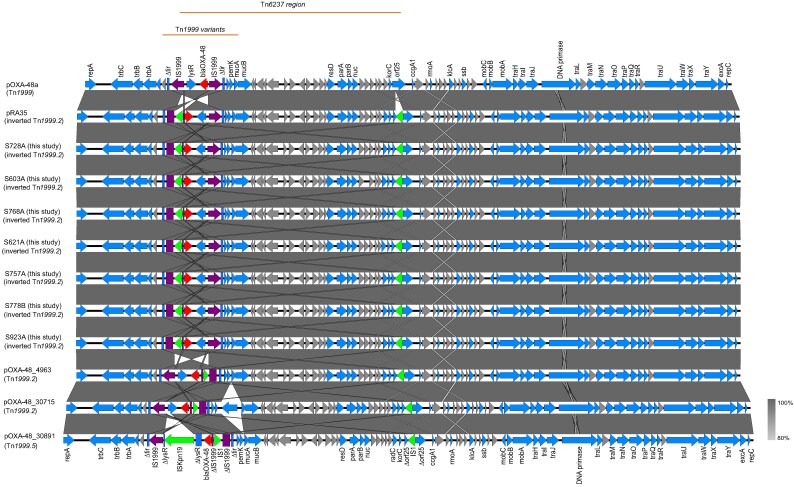
Linear comparison of *bla*_OXA-48_ carrying IncL plasmids from this study (*n* = 7) with reference plasmids pOXA-48a and pRA35, and three plasmids from the Czech outbreak study.^21–23^ The grey shading denotes the homologous regions between the plasmids. The open reading frames for the genes are represented by arrows, with the arrowhead indicating the direction of transcription. The OXA-48 encoding gene is displayed in red colour within the Tn*1999* transposon variants. The square shape represents the truncated genes. IS*1999* insertion sequence elements are coloured purple. IS*1R* and IS*Kpn19* sequence elements are indicated in green. Hypothetical proteins are shown as grey arrows. The boundaries of IS*1R*-based composite transposon Tn*6237* are indicated.

Pairwise comparison with three representative IncL plasmids from a previous Czech OXA-48-like outbreak revealed high similarity with pOXA-48_4963 (KX523900.1), exhibiting 99.8% sequence identity at 100% coverage. pOXA-48_30715 (KX523901.1) shared 99.9% sequence identity at 97% coverage. Both plasmids harboured Tn*1999.2*, but not in the inverted orientation present in isolates from our study. Additionally, pOXA-48_30715 contained an insertion of the *retA* gene upstream of the *mucAB* operon. The third plasmid, pOXA-48_30891 (KX523902.1), exhibited 100% sequence identity at 96% coverage and carried Tn*1999.5*, a variant of Tn*1999.2* defined by the insertion of IS*Kpn19* disrupting the *lysR* gene (Figure 2).^23^

### Analysis of the genetic environment of *bla*NDM-1

**Figure 3.**
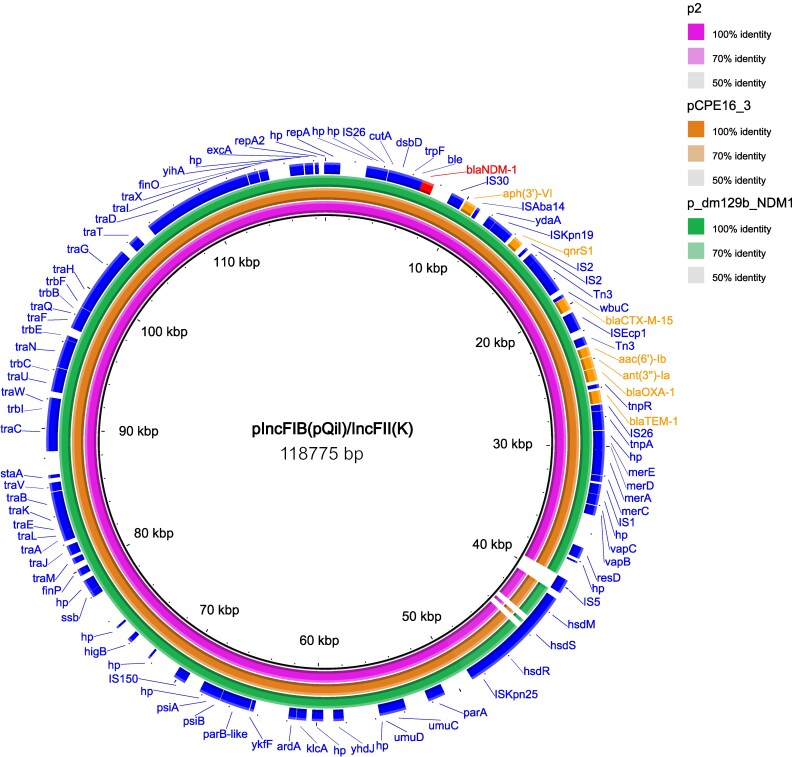
Blast ring image generator (BRIG) visualization of NDM-1 encoding IncFIB(pQil)/IncFII(K) plasmids. The pink-to-green coloured ring indicates the plasmids from different continents: p2, USA (CP009115), pCPE16_3, UK (CP115709) and p_dm129b_NDM1, Bangladesh (CP095596). NDM-1 and other antimicrobial resistance genes are highlighted in red and orange colours, respectively. The outermost blue ring denotes the annotation of the plasmid in the S740A isolate (this study), used as the reference for BRIG comparison.

One *K. pneumoniae* isolate (S740A) of ST866 carried the *bla*_NDM-1_ gene on a multi-replicon IncFIB(pQil)/IncFII(K) plasmid of 118 775 bp. The NDM-1 gene was flanked by an IS30-like element upstream and bleomycin-binding protein (*ble*_MBL_) downstream. The plasmid carried other antimicrobial resistance genes, including *bla*_CTX-M-15_, *bla*_OXA-9,_  *bla*_TEM-1A_, *qnrS1*, *aac(*6′*)-*Ib and *aph(*3′*)-*VI. BLASTn search revealed ≥98% sequence similarity with publicly available plasmids (Figure 3).

## Discussion

Third-generation cephalosporins are widely used in healthcare settings due to their broad-spectrum coverage. However, their overuse imposes selective pressure, driving the rise of ESBL-producing bacterial strains and thus increasing dependence on carbapenems.^31^ In our study, we aimed to determine the prevalence of intestinal carriage of ESBL-producing and/or CRE in hospitalized patients and to investigate the genetic mechanisms underlying carbapenem resistance.

In our study, 30% (239/794) of samples were positive on ESBL-selective agar, a rate considerably higher than those reported in hospitalized patients from Western Europe, such as the Netherlands (4.6%, 188/4119 patients; 2013–22, excluding 2018),^32^ Germany at admission/during hospitalization (12.7%, 169/1334/8.1%, 42/519; 2013–15) and France (17.5%, 97/554; 2014), but comparable to Eastern European countries like Bulgaria 30.2% (94/311; 2015) and Turkey 39.8% (67/168; 2015).^33–36^

In contrast, the prevalence of gut carriage of CRE detected in our study was 3.4%, lower than the ESBL-carriage rate, but still higher compared to hospitalized patients from Western Europe, such as the Netherlands (0.02% for *E. coli* and 0.18% *K. pneumoniae*; 2017–19)^37^ and Denmark (1.5%; 42/2,849, 2016–19),^38^ yet lower than rates reported from Turkey 12.5% (21/168, 2015).^36^ It should be noted that data from Eastern and Central Europe remains limited.

*K. pneumoniae* was the predominant CRE species (92.6%) in our study, with the *bla*_OXA-48_ gene detected in seven isolates. Since the identification of OXA-48 in a hospitalized patient in Turkey in 2001, numerous variants (collectively termed OXA-48-like carbapenemases) have emerged among Enterobacterales, including *E. coli*, *E. cloacae*, and *Citrobacter freundii*.^39^ While these enzymes are detected worldwide, their prevalence shows marked geographical variation. They are endemic in the Middle East, North Africa, the Indian Subcontinent, and several European countries, but account for less than 10% of CPE in Australia, North America and South America.^40^

Unlike the KPC β-lactamase, which is predominantly associated with the epidemic *K. pneumoniae* clone ST258/512, the presence of OXA-48 is not confined to a single sequence types (ST) lineage.^41^ In our study, OXA-48-encoding IncL plasmids occurred in *K. pneumoniae* belonging to four STs (ST11, 45, 54 and 628), whereas ST11 was the most common among non-CP-CRE isolates (*n* = 6/7).

In the Czech Republic, the first detections of Verona integron-encoded metallo-β-lactamase, KPC and NDM carbapenemases were reported between 2009 and 2012, frequently in patients who had either been repatriated from foreign hospitals or had recently travelled abroad.^42–45^ Subsequently, in 2014–15, 24 CPE isolates carrying OXA-48-like carbapenemases were recovered during an outbreak involving seven Czech hospitals, with *K. pneumoniae* accounting for 83.3% (20/24) of the cases.^23^

In the ancestral pOXA-48a plasmid, the *bla*_OXA-48_ gene is carried on a Tn*1999* composite transposon. The IncL plasmids in our dataset, as well as closely related plasmids, carried the Tn*1999.2* variant, characterized by the disruption of an IS*1999* element by the insertion of IS*1R*. Reportedly, the Tn*1999.2* variant has been associated with enhanced OXA-48 expression due to increased imipenem hydrolysis.^46^ The plasmids were also structurally identical to pRA35, which carries the inverted Tn*1999.*2 transposon and the composite transposon Tn*6237*. Interestingly, the chromosomal integration of the *bla*_OXA-48_ gene *via* Tn*6237* has been reported in several countries,^39^ including in *E. coli* and *K. pneumoniae* isolates from the Czech outbreak.^23^ Changing epidemiological trends in the spectrum of carbapenemases in Enterobacterales have been observed in the Czech Republic over the past decade. A significant increase in the prevalence of NDM-1-producing Enterobacterales was reported in 2016,^47^ followed by a notable epidemic spread of KPC producers between 2018 and 2019.^48^ Our data from isolates collected in 2021 reflect the continued dominance of OXA-48 and the ongoing presence of NDM-1. The absence of KPC producers in our dataset, despite their previous upward trend, may indicate localized epidemiological variations within the broader national context. IncX3 plasmids were the dominant driver of NDM-like carbapenemases in the previous Czech study.^47^ We found the *bla*_NDM-1_ gene on an IncFIB(pQil)/IncFII(K) plasmid highly similar to plasmids from clinical *K. pneumoniae* isolates in the USA,^49^ UK^50^ and Bangladesh.

Although vaborbactam and relebactam do not inhibit the hydrolytic activity of OXA-48,^51^ susceptibilities to imipenem/relebactam and meropenem/vaborbactam in the susceptible isolates were primarily determined by the activity of meropenem or imipenem alone, independent of the βLI, as previously described.^52^ However, three meropenem-resistant OXA-48 producers from our study were susceptible to meropenem/vaborbactam. Further research is needed to elucidate how various combinations of mutations in porins, PBPs and efflux pumps overexpression contribute to the development of resistance.

According to the European Society of Clinical Microbiology and Infectious Diseases (ESCMID) guidelines, CRE isolates may still be treated with combination therapy including carbapenems when the meropenem MIC is ≤8 mg/L, although these recommendations do not distinguish between carbapenemase-producing and non-producing isolates.^53^ Similarly, the Infectious Diseases Society of America guidelines support the use of meropenem for ertapenem-mono-resistant strains.^54^ Importantly, carbapenemase production must be excluded before initiating such therapy.

Unexpectedly, 87.5% (7/8) CPE isolates were resistant to cefiderocol, although this drug was not available in the Czech Republic during the sampling period. A previous study indicated that clinically relevant cefiderocol resistance often results from multiple mechanisms, including mutations in iron uptake systems, porin loss and the production of carbapenemases.^55^ Several already described mutations linked to cefiderocol resistance were also found in our isolates, including a cefiderocol-susceptible one with a zone diameter of 23 mm, considered as ATU.^30^

Fosfomycin has reemerged in the treatment of multidrug-resistant infections; however, standardized clinical breakpoints for *K. pneumoniae* remain undefined. Although *fosA* genes are intrinsic to *K. pneumoniae* and were detected in our study, all eight CPE isolates displayed a wild-type phenotype.^56^

In our study, a high proportion (70.4%, 19/27) of CRE isolates were non-CP-CRE. A comparable prevalence rate (60.4%, 157/260) of non-CP-CRE isolates was reported in a 2022 study from Israel.^57^ Consistent with other studies,^57–59^ we observed heterogeneous resistance mechanisms in non-CP-CRE, primarily driven by modifications in porin functions, and co-existing β-lactamase-encoding genes. Importantly, these mechanisms do not uniformly affect *in vitro* susceptibility to all carbapenems.

Among the non-CP-CRE isolates, all were resistant to ertapenem, susceptible to imipenem and 17 of 19 remained susceptible to meropenem. Alterations in porin genes were observed in all ertapenem-resistant isolates, consistent with previous studies showing that ertapenem is particularly affected by resistance mechanisms such as porin loss compared to other carbapenems.^60^ In two meropenem-resistant isolates, hybrid genome assemblies revealed an increased copy number of the *bla*_CTX-M-15_ gene, facilitated by IS*Ecp1* elements. The combination of gene amplification and porin modifications could confer resistance to carbapenems such as ertapenem and meropenem; however, this hypothesis requires experimental confirmation. Additionally, in one *E. coli* isolate, an IS26-bound transposon carrying *bla*_CTX-M-27_ was inserted into the *ompC* gene, resulting in its disruption. These findings are consistent with the work of Shropshire *et al.*, where both IS*26* and/or IS*Ecp1*-mediated ESBL amplification and porin disruption contributed to the emergence of carbapenem resistance, particularly ertapenem and/or meropenem.^7,8^

According to current infection control recommendations, patients colonized or infected with CRE should ideally be managed in single rooms with dedicated bathroom facilities to prevent cross-transmission. In inter-facility transfers, it is essential that the patient’s CRE status be clearly communicated to the receiving institution to ensure continuity of appropriate infection control measures.^61^

A limitation of this study is that not all isolates underwent long-read sequencing to determine the exact number of *bla*_CTX-M-15_ gene copies, and gene expression levels were not assessed to evaluate the impact of copy number variation. Further experimental studies are warranted to functionally validate the identified mutations and confirm their contribution to carbapenem resistance.

### Conclusion

In conclusion, a 30% intestinal carriage prevalence of ESBL-producing Enterobacterales and a 3.4% carriage prevalence of CRE were observed. Long-read sequencing revealed plasmid-mediated OXA-48 dissemination and *bla*_CTX-M-15_ gene amplification, demonstrating the added value of this technology for antimicrobial resistance monitoring.

## Supplementary Material

dlaf254_Supplementary_Data

## Data access

All raw sequence data from this study were submitted to the NCBI Sequence Read Archive under accession number BioProject ID PRJNA1258574. The whole-genome and plasmid sequences from the eight carbapenemase producers and selected non-carbapenemase-producing isolates S613A, S653A, S822B, S795C, S800B and S931B (accession numbers provided in Tables S1 and S2) have been deposited in GenBank.

## Supplementary data

Tables S1–S3 are available as Supplementary data at *JAC-AMR* Online.
